# Assessing the Contribution of Relative Macrophage Frequencies to Subcutaneous Adipose Tissue

**DOI:** 10.3389/fnut.2021.675935

**Published:** 2021-05-31

**Authors:** Marianthi Kalafati, Michael Lenz, Gökhan Ertaylan, Ilja C. W. Arts, Chris T. Evelo, Marleen M. J. van Greevenbroek, Ellen E. Blaak, Michiel Adriaens, Martina Kutmon

**Affiliations:** ^1^Deparment of Human Biology, NUTRIM School of Nutrition and Translational Research in Metabolism, Maastricht University, Maastricht, Netherlands; ^2^Maastricht Centre for Systems Biology, Maastricht University, Maastricht, Netherlands; ^3^Institute of Organismic and Molecular Evolution, Johannes Gutenberg University of Mainz, Mainz, Germany; ^4^Preventive Cardiology and Preventive Medicine—Center for Cardiology, University Medical Center of the Johannes Gutenberg-University Mainz, Mainz, Germany; ^5^Unit Health, Flemish Institute for Technological Research, Antwerp, Belgium; ^6^Department of Epidemiology, CARIM School for Cardiovascular Diseases, Maastricht University, Maastricht, Netherlands; ^7^Department of Bioinformatics-BiGCaT, NUTRIM School of Nutrition and Translational Research in Metabolism, Maastricht University, Maastricht, Netherlands; ^8^Department of Internal Medicine, CARIM School for Cardiovascular Diseases, Maastricht University, Maastricht, Netherlands

**Keywords:** subcutaneous adipose tissue, low-grade inflammation, lipid metabolism, macrophages, cell-type composition, computational deconvolution, publicly available data

## Abstract

**Background:** Macrophages play an important role in regulating adipose tissue function, while their frequencies in adipose tissue vary between individuals. Adipose tissue infiltration by high frequencies of macrophages has been linked to changes in adipokine levels and low-grade inflammation, frequently associated with the progression of obesity. The objective of this project was to assess the contribution of relative macrophage frequencies to the overall subcutaneous adipose tissue gene expression using publicly available datasets.

**Methods:** Seven publicly available microarray gene expression datasets from human subcutaneous adipose tissue biopsies (*n* = 519) were used together with TissueDecoder to determine the adipose tissue cell-type composition of each sample. We divided the subjects in four groups based on their relative macrophage frequencies. Differential gene expression analysis between the high and low relative macrophage frequencies groups was performed, adjusting for sex and study. Finally, biological processes were identified using pathway enrichment and network analysis.

**Results:** We observed lower frequencies of adipocytes and higher frequencies of adipose stem cells in individuals characterized by high macrophage frequencies. We additionally studied whether, within subcutaneous adipose tissue, interindividual differences in the relative frequencies of macrophages were reflected in transcriptional differences in metabolic and inflammatory pathways. Adipose tissue of individuals with high macrophage frequencies had a higher expression of genes involved in complement activation, chemotaxis, focal adhesion, and oxidative stress. Similarly, we observed a lower expression of genes involved in lipid metabolism, fatty acid synthesis, and oxidation and mitochondrial respiration.

**Conclusion:** We present an approach that combines publicly available subcutaneous adipose tissue gene expression datasets with a deconvolution algorithm to calculate subcutaneous adipose tissue cell-type composition. The results showed the expected increased inflammation gene expression profile accompanied by decreased gene expression in pathways related to lipid metabolism and mitochondrial respiration in subcutaneous adipose tissue in individuals characterized by high macrophage frequencies. This approach demonstrates the hidden strength of reusing publicly available data to gain cell-type-specific insights into adipose tissue function.

## Introduction

The adipose tissue is an endocrine and immunologically active organ, that combined with its insulative and dynamic energy storage functions affects the regulation of systemic energy and inflammatory homeostasis (1). It consists of two main components, a heterogeneous cellular population and an extracellular matrix (2). The most abundant cell type in adipose tissue is the adipocyte. Other cell types are also present including preadipocytes, mesenchymal stem cells, fibroblasts, endothelial cells, and many immune cells, including adipose tissue macrophages (ATMs) (3).

In adipose tissue, there are resident macrophages and monocyte-derived macrophages, which collectively are called ATMs. In general, macrophages are phagocytes that preserve tissue homeostasis by finding and removing cell debris, pathogens, and apoptotic or necrotic cells. ATMs are present in lean and obese adipose tissues, involved in the regulation of adipogenesis and angiogenesis (4). Macrophages in lean humans constitute around 5% of the cells in adipose tissue, whereas during obesity they constitute up to 50% of all adipose tissue cells (5). Chronic metabolic diseases promote macrophage infiltration often leading to adipose tissue inflammation (4–7), characterized by increased secretion of adipokines and cytokines into the systemic circulation, which may be associated with hepatic and peripheral insulin resistance (8).

The area of immunometabolism aims to understand how changes in intracellular metabolic pathways in immune cells alter their function and, how immune cells employ tissue metabolism in adaptation to environmental changes. Understanding therefore not only the contribution of macrophages and other immune cells to adipose tissue dysfunction and plasticity but also the interplay with adipocytes, adipokines, adipose tissue-secreted cytokines, and other nonadipose tissues, can offer insights on how to control immunometabolism in health and disease. Describing therefore adipose tissue cell-type composition is particularly important. In general, tissue cell-type composition can be estimated with common experimental methods (e.g., flow cytometry) or computational deconvolution [e.g., CIBERSORT (9)]. Experimental methods can be costly and are often logistically impractical for large human cohort studies. Researchers can overcome these limitations by using computational methods to estimate cell type composition of complex tissues from their gene expression profiles (9).

In this paper, our analysis assesses the contribution of macrophage frequencies to the overall SAT gene expression. We used the computational algorithm TissueDecoder (10) to infer the SAT cell-type composition from publicly available, gene expression microarray datasets. Furthermore, we divided our samples based on their relative macrophage frequencies and set out to study the relation between interindividual differences in relative macrophage frequencies and transcriptional differences in metabolic and inflammatory pathways within the SAT.

## Materials and Methods

### Deconvolution of Adipose Tissue Cell Types by TissueDecoder

The TissueDecoder (10) framework uses CIBERSORT (9) to estimate the cellular composition of adipose tissue samples from their whole tissue gene expression profiles. TissueDecoder provides a novel signature matrix (AT21) that includes highly relevant cell types for adipose tissue, based on publicly available data from the Affymetrix Human U1333 Plus 2.0 microarray. Briefly, the AT21 reference dataset is generated using single cell-type gene expression data from 21 different cell types from 204 samples that were collected from publicly available datasets in the Gene Expression Omnibus (GEO) (11) and ArrayExpress (12) databases. The raw data (CEL files) were preprocessed with the Affymetrix Power Tools using the robust multiarray average normalization method as described in the original publication by Lenz et al. (10). CIBERSORT together with the AT21 matrix were used to deconvolute the 779 samples, thus determining the relative frequencies of 21 cell types. Additional information on the probe selection criteria can be found in the original publication (10).

In our analysis, we initially included 616 human subcutaneous adipose tissue (SAT) samples from eight studies [GSE27916 (13), GSE20950 (14), GSE27657 (15), GSE41168 (16), GSE66159 (17), E-MTAB-1895 (18), GSE26637 (19), GSE27949 (20)], combined into one SAT dataset. As we wanted to adjust for sex in the linear regression models, samples without sex information were excluded, thus 583 samples and seven studies remained in our analysis (GSE27949 was excluded, as 33 samples did not have sex information). Furthermore, 64 samples from studies with multiple time points, e.g., samples after intervention, were excluded (54 samples from GSE41168 and 10 samples from GSE66159). Finally, 519 SAT samples and seven studies remained.

### Filtering on Absolute Expression Level

The seven studies with the 529 SAT samples comprised 54,675 probes. Firstly, 11,953 probes without gene identifiers were removed, thus 42,722 remained. The probes with the duplicated gene identifiers (19,622 probes) were summarized into one unique gene identifier (per duplicated probe) calculating the medians across samples. The probes with the highest median expression were kept resulting in 23,100 unique genes. Furthermore, the median expression of the Y chromosome genes in female subjects was defined as the gene expression detection threshold. Y chromosome genes are not expressed in female subjects and hence provide a measure of background noise. Genes with a median expression below the computed threshold (3.1 on a log_2_ scale) were removed. Finally, from 23,100 unique genes, 4,056 genes were expressed below background level and therefore removed, resulting in 19,043 unique genes considered for downstream analysis. The probe identifiers were additionally mapped to the *Homo sapiens* GRCh38 assembly in Ensembl (21) through the BioMart (22) library in R (v3.6.3).

### Grouping Subjects Based on Relative Macrophage Amount

The SAT dataset individuals were divided into four groups based on their relative adipose tissue macrophage frequencies. Individuals with no detectable frequencies of macrophages were defined as M0 group. The remaining subjects were divided into tertiles, defined as M1 (%0–1), M2 (%1–2), and M3 (%2–25).

### Statistical Analysis

#### Participant Characteristics

We assessed the differences in the cell-type frequencies between groups using a Wilcoxon rank sum test. Multiple testing correction was performed by applying the Benjamini and Hochberg method on the *p*-values, to control the false-discovery rate (FDR). The threshold for statistical significance for nominal and FDR *p*-values was set at *p* < 0.05.

#### Differential Gene Expression Analysis

We focused our analysis on the extremes (M3 vs. M0). Differential gene expression analysis was implemented using *limma* (v3.42.2) (23). To assess the effect of adjusting for cell-type composition, two linear models were implemented for the differential gene expression analysis, (i) adjusting for sex and study (model 1) and (ii) additionally adjusting for differences in cell-type composition (20 cell types excluding macrophages). All significant genes (nominal *p* < 0.05) were divided into up- (nominal *p* < 0.05 and log_2_ fold change > 0.26) and downregulated (nominal *p* < 0.05 and log_2_ fold change < −0.26).

#### Gene Ontology Analysis

Gene Ontology (GO) analysis was performed using *clusterProfiler* (v3.14.3) (24). All up- and downregulated genes were included separately to provide direction for the involved biological processes. The organism *Homo sapiens* and the ontology biological process were used. The gene-mapping database was set to “ENSEMBL.” Finally, the overrepresentation results were simplified using the function *simplify* filtering on FDR-*p* < 0.05 and similarity cut-off 0.7.

#### Pathway Analysis

Pathway analysis was performed with rWikiPathways (v1.6.1) (25). We used the curated human pathway collection (20200610). An overrepresentation analysis was performed with the microarray dataset. Pathways were significantly changed and considered for the analysis when FDR-*p* < 0.05.

#### Network Analysis

The top 10 GO biological processes for the up- and downregulated genes were exported as a process-gene network using the function *cnetplot* from clusterProfiler. The nodes in the networks represented genes and biological processes. They were connected by an edge when a gene was involved in the process. The networks were imported in Cytoscape (v3.8.0) (26) with the function *createNetworkFromIgraph via* the RCy3 (v2.6.3) (27). Only the relevant differentially expressed genes for the enriched processes were shown in the networks. The pie chart visualization for the process nodes showed how many genes were associated with the biological process in total and their overall expression patterns. The differential gene expression was visualized using a color gradient for the node fill color in the networks.

#### Sensitivity Analysis

We performed a sensitivity analysis excluding study GSE27916 (with 375 participants) to check whether the results we obtained were mainly driven by this dataset, as a large proportion of the samples were included in our SAT dataset. Likewise, we performed an additional sensitivity analysis excluding study E-MTAB-1895 (with 52 participants) which consisted of twins to evaluate if our results were driven by the nonindependence of these participants. Differential gene expression and GO analysis was performed as described in the “Materials and Methods” section above.

### Results

#### Dataset Characteristics

The human SAT dataset was composed of 519 samples and seven studies, all publicly available. Briefly, study GSE20950 (14) contained expression data from BMI-matched obese cohort individuals that were either insulin sensitive or insulin resistant. Study GSE26637 (19) contained expression data from obese insulin sensitive and insulin-resistant females. Study GSE27657 (15) contained expression data of individuals undergoing surgery in the thyroid region. Study GSE27916 (13) contained expression data from the Swedish Obese Subjects Sib-Pair offspring cohort. Furthermore, study GSE41168 (16) contained expression data from nonobese individuals with normal glucose tolerance. Study GSE66159 (17) contained expression data from overweight or obese females at moderately increased risk of breast cancer. Finally, the E-MTAB-1895 (18) contained expression data from young adult obesity-discordant monozygotic twin pairs.

#### Participant Characteristics

The SAT dataset individuals were divided into four groups based on their relative adipose tissue macrophage frequencies. Individuals with no detectable frequencies of macrophages were defined as M0 group. The remaining subjects were divided into tertiles according to the percentage (frequency) of macrophages in the tissue, defined as M1 (%0–1), M2 (%1–2), and M3 (%2–25). Since the studies included in our analysis were publicly available, only a limited amount of phenotypic information was available. Briefly, in M0, there were 115 participants (82 women), in M1 were 135 participants (106 women), in M2 were 135 participants (94 women), and in M3 were 134 participants (90 women) (Supplementary Table 4). Thus, the groups did not differ with respect to female/male distribution, and in all groups, females were more prevalent. Additionally, from the M0 to M3 groups, based on the available characteristics, obesity and insulin resistance seem to worsen (Supplementary Figures 1, 2). Detailed phenotypic participant characteristics are reported separately for each group and study in Supplementary Table 1.

#### High Macrophage Frequencies Are Associated With Higher Adipose Stem Cell and Lower Subcutaneous Adipocyte Frequencies in SAT

**Figure 1:**
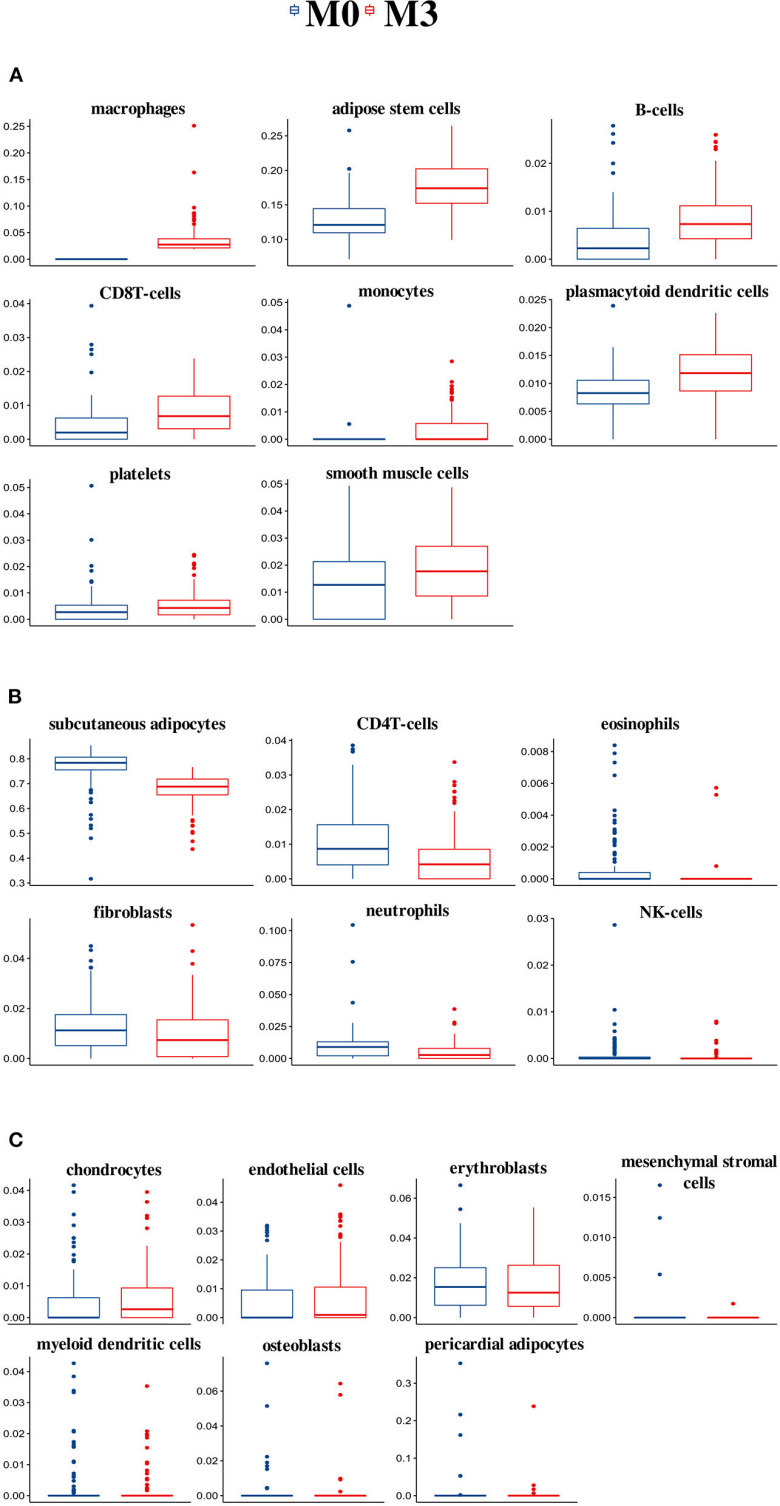
Cell-type composition and differences between the M0 and M3 groups of the 21 cell types estimated by TissueDecoder in the SAT dataset. Compared with the M0 group, individuals in M3 had higher relative frequencies of eight cell types **(A)**, lower relative frequencies of six cell types **(B)**, and no significant differences for seven cell types **(C)**.

We focused our analysis on the extremes (M3 vs. M0). Compared with the M0 group, individuals in M3 had higher relative frequencies of eight cell types, namely macrophages, adipose stem cells, B cells, CD8T cells, monocytes, plasmacytoid dendritic cells, platelets, and smooth muscle cells (Figure 1A). Six cell types had lower relative frequencies in the M3 group compared with the M0 group. Those were the subcutaneous adipocytes, CD4T cells, eosinophils, fibroblasts, neutrophils, and NK-cells (Figure 1B). Finally, for seven cell types, there were no significant differences between the M0 and M3 group (Figure 1C). Those were the chondrocytes, endothelial cells, erythroblasts, mesenchymal stromal cells, myeloid dendritic cells, osteoblasts, and pericardial adipocytes. Information on cell-type composition and differences (*p*-values) for the M1 and M2 group comparisons is provided in Supplementary Table 2.

#### SAT Transcriptome Differences Between Groups With High and Low Macrophage Frequencies

**Figure 2:**
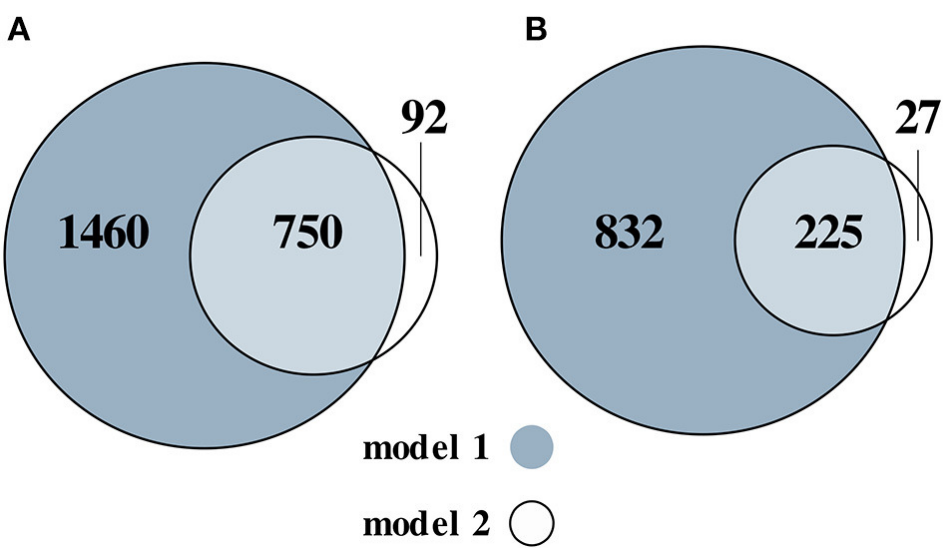
Venn diagram of the numbers of the upregulated **(A)** and downregulated **(B)** genes, for the M3 vs. M0 group comparison. Two models were used: adjusted for sex and (model 1) and additionally adjusted for differences in cell-type composition (model 2).

Next, we assessed the SAT transcriptome for the M3 vs. M0 group comparison. The M0 group was used as a reference. We identified 2,210 upregulated (nominal *p* < 0.05 and log_2_ fold change > 0.26) genes after adjusting for sex and study (model 1) and 842 after additionally adjusting for differences in cell-type composition (model 2) (Figure 2A; Supplementary Table 3). We identified 1,057 downregulated (nominal *p* < 0.05 and log_2_ fold change < −0.26) genes in model 1 and 252 in model 2 (Figure 2B; Supplementary Table 4).

#### GO Enrichment Analysis Between High and Low M Groups

To gain further insight into the differentially expressed SAT transcriptome after adjusting for sex and study (model 1) and additionally adjusting for cell-type composition differences (model 2), GO analysis was performed for the M3 vs. M0 comparison for the up- and downregulated genes, separately, to provide direction of the biological processes. All 2,210 upregulated and 1,057 downregulated genes from model 1 and all 842 upregulated and 252 downregulated genes from model 2 were used. The analysis resulted in 252 significant biological processes for the upregulated genes (Supplementary Table 5) and 85 for the downregulated genes (Supplementary Table 6) for model 1 and in 248 significant biological processes for the upregulated genes (Supplementary Table 7) and 46 for the downregulated genes (Supplementary Table 8) for model 2. Additionally adjusting for differences in cell-type composition did not materially alter the results, therefore the analysis was continued with model 1 (adjusting for sex and study).

#### Increased Inflammatory SAT Gene Expression Associated With High Macrophage Frequencies

**Figure 3:**
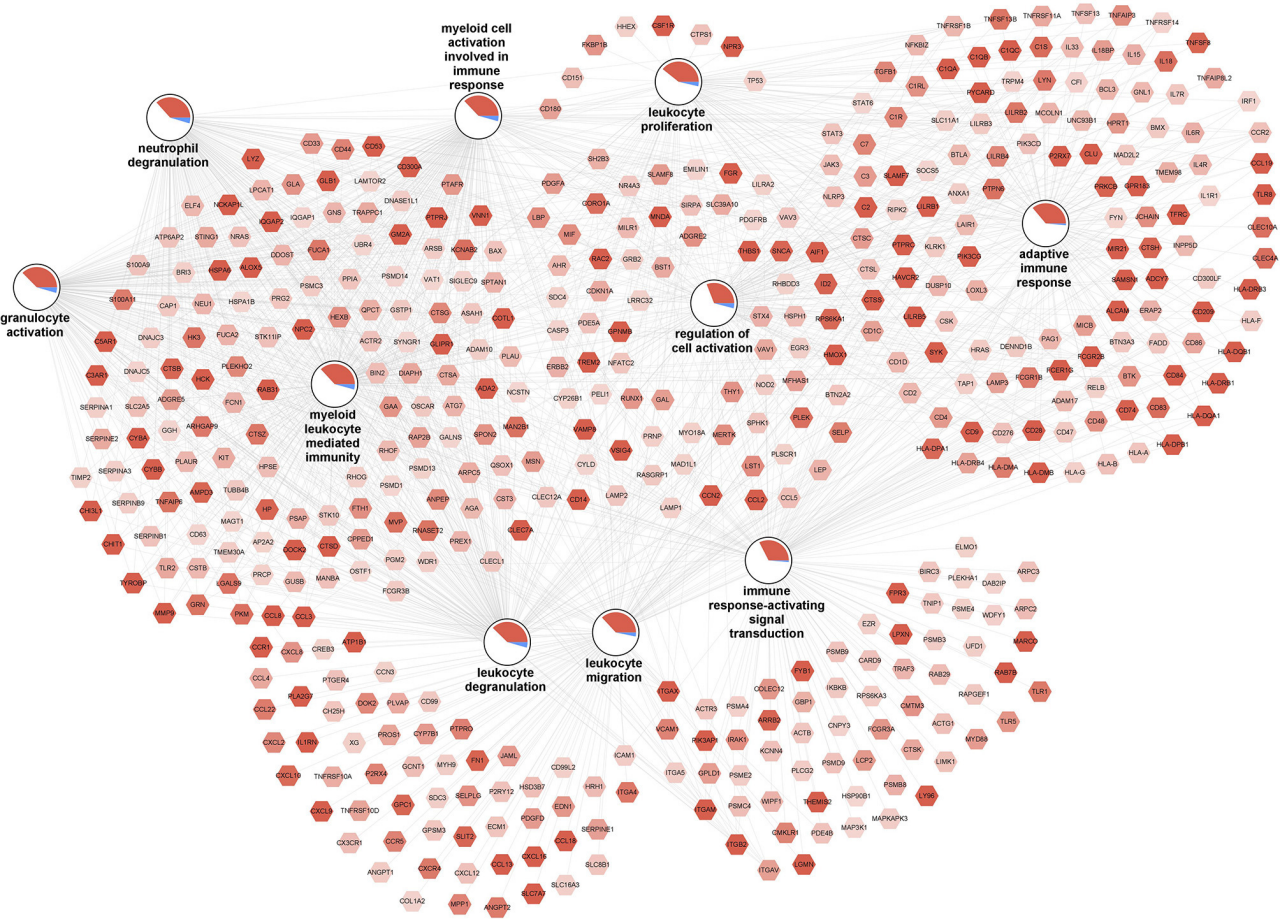
Increased inflammation associated with high macrophage frequencies in SAT. The gene-process network illustrates the top 10 GO biological processes enriched for upregulated genes for the M3 vs. M0 group comparison. Upregulated gene nodes are visualized as hexagons and process nodes as circles. The edges in the network show the involvement of genes in biological processes to which they are connected to. The differential gene expression is visualized on the gene nodes using a color gradient from blue (downregulated) over white (not changed) to red (upregulated). The overall distribution of gene expression changes for genes associated with a biological process is visualized using a pie chart divided into upregulated, downregulated, and not significantly changed genes.

The top 10 enriched biological processes (FDR-*p* < 0.05) for the upregulated genes were selected and combined into a network (Figure 3). The network illustrates a gene-process network in which the nodes represent upregulated genes and enriched processes, and they are connected by edges which show that the genes are directly involved in the biological process. The overall expression patterns are shown in the pie chart visualization of the process nodes. Only the 472 upregulated genes in the top 10 processes are added in the network visualization. In additional analyses, we performed a pathway overrepresentation analysis with all upregulated genes for the M3 vs. M0 group comparison. The analysis identified 55 significantly changed pathways (FDR-*p* < 0.05; Supplementary Table 9). Among those pathways were the “human complement system (WP2806),” “regulation of toll-like receptor signaling pathway (WP1449),” “chemokine signaling pathway (WP3929),” “type II interferon signaling (IFNG) (WP619),” “focal adhesion (WP4172),” “oxidative damage (WP3941),” “AGE/RAGE pathway (WP2324),” and “VEGFA-VEGFR2 signaling pathway (WP3888).” SAT of individuals with high numbers of macrophages was characterized by higher expression of key genes involved in complement activation (e.g., *C1QA, C1QB*, and *C1QC*), chemotaxis (e.g., *CCL2, CCL3*, and *CCL5*), and adhesion molecules (e.g., *ITGB2, VCAM1*, and *ICAM1*); major histocompatibility complex (MHC) class I (e.g., *HLA-A* and *HLA-B*) and II (e.g., *HLA-DPA1, HLA-DRB1*, and *HLA-DQA1*). Furthermore, it was characterized by higher expression of genes involved in extracellular matrix (ECM) organization [e.g., collagens, metalloproteinase domain-containing protein (ADAMs), matrix metalloproteases (MMPs), and tissue inhibitor of metalloproteinases (TIMPs)], angiogenesis (e.g., *VEGFB, SERPINE1, ANGPTL4*), and oxidative stress (e.g., *CYBB* and *CYBA*). These data reveal activation of inflammatory pathways and an overrepresentation of inflammatory GO biological processes, indicating that high macrophage frequencies in the SAT associates with increased SAT inflammatory gene expression.

#### Dysfunctional Lipid and Glucose Metabolism, Mitochondrial Respiration, and BCAA Catabolism Associated With High Macrophage Frequencies in SAT

**Figure 4:**
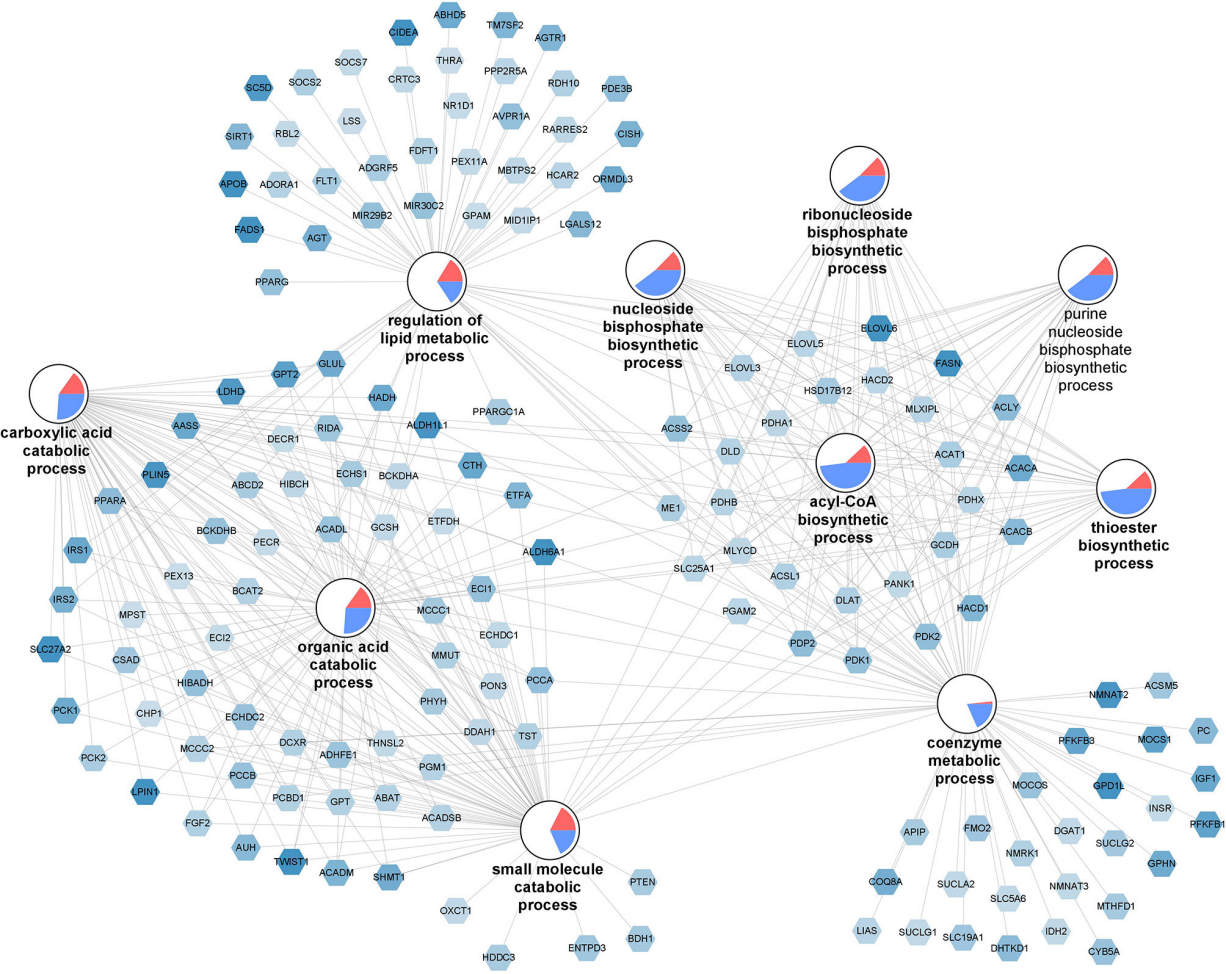
Dysfunctional lipid and glucose metabolism, mitochondrial respiration, and BCAA catabolism associated with high macrophage frequencies in SAT. The gene-process network illustrates the top 10 GO biological processes enriched for downregulated genes for the M3 vs. M0 group comparison. Downregulated gene nodes are visualized as hexagons and process nodes as circles. The edges in the network show the involvement of genes in biological processes to which they are connected to. The differential gene expression is visualized on the gene nodes using a color gradient from blue (downregulated) over white (not changed) to red (upregulated). The overall distribution of gene expression changes for genes associated with a biological process is visualized using a pie chart divided into upregulated, downregulated, and not significantly changed genes.

The top 10 enriched biological processes (FDR-*p* < 0.05) for the downregulated genes were selected and combined into a network (Figure 4). The network illustrates a gene-process network in which the nodes represent downregulated genes and enriched processes, and they are connected by edges which show that the genes are directly involved in the biological process. The overall expression patterns are shown in the pie chart visualization of the process nodes. Only the 157 downregulated genes in the top 10 processes are added in the network visualization. Following the GO analysis, we performed pathway analysis. The analysis revealed 12 significantly changed pathways (Supplementary Table 10). Among those pathways were, “fatty acid biosynthesis (WP357),” “amino acid metabolism (WP3925),” and “adipogenesis (WP236).” Key genes involved in lipid and glucose metabolism [glucose transporters e.g., *SLC27A2* (also known as *FATP2*), *IRS1* and *IRS2, LPIN1*], fatty acid metabolism (e.g., *ACACA, FASN*) and fatty acid oxidation (e.g., *PPARA, PPARG*), lipogenesis (e.g., *ACLY, ELOVL6, FADS1, FADS2*), adipogenesis (e.g., *KLF15, FOXO1, IGF1, TWIST1*), and angiogenesis (e.g., *VEGFA, FGF2*) were significantly downregulated in individuals with high macrophage frequencies. Furthermore, key genes involved in mitochondrial respiration [e.g., *PPARG* and *PGC1A* (also known as *PPARGC1A*), *COX7C* and *COX14, NDUFA8, NDUFB5* and *NDUFS4, ATP11B* and *ATP8A2*] were significantly downregulated in individuals with high macrophage frequencies. On the same direction, key genes involved in degradation of all BCAAs, namely isoleucine, leucine, and valine (e.g., *BCKDHB*), and those specific to isoleucine (e.g., *PCCA* and *PCCB*), leucine (e.g., *AUH*), and valine (e.g., *HIBADH* and *ALDH6A1*) were significantly downregulated in the individuals with high macrophage frequencies. Collectively, these data reveal a decreased lipid and glucose metabolism, mitochondrial respiration, and BCAA catabolism associated with high macrophage frequencies in SAT.

#### Sensitivity Analysis

We performed a sensitivity analysis excluding study GSE27916 (with 375 participants) to check whether the results we obtained were mainly driven by this dataset, as a large proportion of the samples were included in our SAT dataset. Likewise, we performed an additional sensitivity analysis excluding study E-MTAB-1895 (with 52 participants) which consisted of twins to evaluate if our results were driven by the nonindependence of these participants. Differential gene expression and GO analysis was performed. Exclusion of these datasets did not materially alter the results (Supplementary Tables 11–18).

### Discussion

#### Methodology and Benefit of the Analytic Approach

Our analysis demonstrated the substantial benefit of integrating publicly available datasets in combination with the algorithm TissueDecoder, in assessing the contribution of macrophage frequencies to the overall subcutaneous adipose tissue gene expression. TissueDecoder provides a signature matrix with cell types relevant for obesity, type 2 diabetes, insulin resistance, and other metabolic abnormalities. We used the inferred cell-type composition from SAT Affymetrix microarray data to identify individuals with high and low macrophage frequencies, as macrophage content in adipose tissue predicts the risk for metabolic disease. We showed the relative frequencies of adipocytes to be lower and the relative frequencies of adipose stem cells to be higher in individuals characterized by high macrophage frequencies. Notably, TissueDecoder estimates 20 more cell types across three additional depots (omental, epicardial, and pericardial adipose tissue). The proposed approach can therefore be used in a similar manner to define other groups of interest (e.g., subcutaneous adipocytes or adipose stem cells) in investigating adipose tissue metabolism.

#### Biological Implications of Our Findings

We observed lower frequencies of adipocytes and increased frequencies of adipose stem cells in the individuals with high macrophage frequencies compared with those with low ones. Failure of adipocyte differentiation has been associated with reduced expandability of adipose tissue, contributing to adipose tissue inflammation, systemic inflammation, lipid overflow, and insulin resistance (8, 28, 29), while low numbers of adipocytes may be associated with impaired metabolic health (30). In line, previous studies have showed that obese-derived adipose stem cells have decreased differentiation, migration, and angiogenic capabilities (31, 32) attributed to differences in the anatomical distribution of adipose tissue (33) or decrease in adipose stem cells in obese humans (34, 35) and mice (35, 36). Based on the available phenotypic data, obesity and insulin resistance status seem to worsen in individuals characterized by high macrophage frequencies, lower percentage of adipocytes, and higher percentage of adipose stem cells. Overall, our data suggest that those individuals are characterized by an impaired preadipocyte differentiation and a limited capacity for hyperplasia, which may contribute to adipose tissue dysfunction, insulin resistance, and a generally unfavorable metabolic profile.

We observed that adipose tissue of individuals characterized by high SAT macrophage frequencies, exhibited the expected increased inflammatory gene expression profile accompanied by decreased gene expression in pathways related to lipid metabolism, mitochondrial respiration, and BCAA catabolism. In individuals characterized by high macrophage frequencies, we observed a higher expression of genes involved in inflammatory processes (e.g., chemotaxis, complement activation), ECM remodeling, oxidative stress, and angiogenesis. Macrophage amount but also change in their localization is associated with increased chemokine expression and increased inflammation in the adipose tissue (6, 37, 38), obesity (5, 7) and insulin resistance (39). Furthermore, increased expression of complement components has been previously associated with an increased inflammatory profile in SAT in obesity, suggesting complement involvement in the clearance of apoptotic debris in the adipose tissue (40). Next, upregulation of genes related to ECM organization in SAT has been associated with a reduced adipose tissue expansion capacity and dysfunctional adipose tissue in obesity (41, 42) and with hepatic insulin resistance (43). Alcala et al. suggested that oxidative stress plays an important role in ECM remodeling and therefore metabolic regulation in mice (44). On that note, Van den Bossche et al. (45) reported that oxidative stress promotes M1 macrophage polarization, while a plethora of studies have corroborated that adipose tissue undergoes increased oxidative stress due to obesity induced by overnutrition (46, 47). Finally, the vascular endothelial growth factors (VEGFs) are key factors in angiogenesis and adipose tissue remodeling and *VEGF* has been reported to be a chemotactic for macrophages (48). In line with our results Lu et al. reports *VEGFB* upregulation was associated to *VEGFA* downregulation, as a compensatory mechanism that leads to brown-like white adipose tissue differentiation (49). Collectively, our data show that individuals characterized by high macrophage frequencies have higher expression of genes involved in inflammatory processes and ECM remodeling, suggesting increased SAT inflammation.

Moreover, we observed lower expression in genes involved in lipid and glucose metabolism, mitochondrial respiration and BCAA catabolism in the individuals with high macrophage composition. Similar observations have been previously reported with obesity and/or insulin resistance (19, 50–53). Especially during hyperinsulinemic conditions, the lower expression of mitochondrial pathways is an important finding as it could reflect a regulatory defect, that potentially further advances the pathogenesis of insulin resistance (19). Nilsson et al. reported genes involved in adipose tissue lipogenesis strongly downregulated in the SAT from monozygotic twins discordant for type 2 diabetes (54). During the development of obesity and insulin resistance, the adipose tissue can reach a flexing point while adipocytes reduce their ability to synthesize additional fatty acids or triglycerols, resulting in decreased lipogenesis (55, 56). Furthermore, we found lower expression of genes involved in amino acid metabolism. Wiklund et al. (57) reported lower SAT expression of genes related to BCCA catabolism and mitochondrial energy metabolism along with increased expression of genes involved in inflammatory processes in insulin resistant subjects. Collectively, our data show that individuals characterized by high macrophage frequencies have lower expression of genes involved in lipid metabolism and mitochondrial respiration, suggesting adipose tissue dysfunction and impaired adipocyte differentiation, enhanced by the increased SAT inflammation.

#### Strengths, Limitations, and Future Directions

Our analysis exhibits the substantial benefit of combining and reusing publicly available data. Additionally, it allows assessing the contribution of macrophage frequencies to the overall SAT gene expression. Furthermore, this type of analysis allows researchers to investigate other cell types potentially involved in the dysregulation of adipose tissue metabolism. Naturally, our analysis comes with shortcomings; publicly available datasets do not provide sufficient information on phenotypic measures (e.g., BMI, HOMA-IR, or age) that could be adjusted for in the linear regression analysis. Finally, a context-specific signature matrix from isolated cells from the tissue of interest could further improve the computational predictions of the cell-type composition (10). Additionally, for studying adipose tissue inflammation, identifying signatures for macrophage subtypes could be of interest.

In conclusion, we have shown the additive value of integrating publicly available datasets in combination with the useful application of cell-type composition in SAT gene expression. The contribution of macrophage frequencies and other cell types to adipose tissue dysfunction and plasticity can offer insights in modulating human health and disease by providing targets and biomarkers for more personalized risk classification in the prevention and treatment of obesity and its complications. We hypothesized that increased macrophage and adipose stem cell percentage and the decreased percentage of adipocytes reflects adipose tissue inflammation and impaired preadipocyte differentiation, possibly reflective of a limited capacity for hyperplasia, and adipose tissue dysfunction that contributes to an unfavorable metabolic profile. Further studies should investigate whether classifying individuals based on their M1 or M2 macrophage profile has a similar effect in SAT gene expression profiles.

## Data Availability Statement

The original contributions presented in the study are included in the article/Supplementary Material, further inquiries can be directed to the corresponding author/s.

## Ethics Statement

Ethical review and approval was not required for the study on human participants in accordance with the local legislation and institutional requirements. Written informed consent for participation was not required for this study in accordance with the national legislation and the institutional requirements.

## Author Contributions

MKa wrote the manuscript and performed the transcriptomics and statistical analyses. IA, EB, MG, MA, and MKu supervised the transcriptomics and/or statistical analyses and contributed to the conception or design of the current work. ML and GE designed the computational deconvolution algorithm, selected, and pre-processed the publicly available SAT datasets. MKa, ML, GE, IA, CE, EB, MG, MA, and MKu interpreted the data and/or revised the manuscript. All authors approved the manuscript.

## Conflict of Interest

The authors declare that the research was conducted in the absence of any commercial or financial relationships that could be construed as a potential conflict of interest.

## Supplementary Material

Supplementary Figure 1 Mosaic plot representing the participant's obesity status per M group. Based on the available characteristics, obesity seems to worsen from the M0 to M3 groups.

Supplementary Figure 2 Mosaic plot representing the participant's insulin-resistant status per M group. Based on the available characteristics, insulin resistance seems to worsen from the M0 to M3 groups.

Supplementary Table 1 The participant characteristics in the SAT dataset from 519 individuals that were divided in four groups. Individuals with no detectable amount of macrophages were defined as M0 group.

Supplementary Table 2 Cell-type composition and differences within the M groups of the 21 cell-types estimated by TissueDecoder for the SAT dataset.

Supplementary Table 3 Significantly upregulated genes for the M3 vs. M0 group comparison for models 1 and 2. Model 1 adjusts for sex and study and model 2 additionally adjusts for differences in cell-type composition. The log_2_ fold changes, nominal, and FDR *p*-values are shown.

Supplementary Table 4 Significantly downregulated genes for the M3 vs. M0 group comparison for models 1 and 2. Model 1 adjusts for sex and study and model 2 additionally adjusts for differences in cell-type composition. The log_2_ fold changes, nominal, and FDR *p*-values are shown.

Supplementary Table 5 Significant biological processes (upregulated genes) for the M3 vs. M0 group comparison adjusted for sex and study (model 1). The column significant genes shows the number of significant genes identified from our dataset for this specific GO term. The total # genes shows the total number of genes associated with that specific GO term. Nominal *p*-values and false discovery rate (FDR) *p*-values for the GO terms are also presented.

Supplementary Table 6 Significant biological processes (upregulated genes) for the M3 vs. M0 group comparison adjusted for sex and study (model 1). The column significant genes shows the number of significant genes identified from our dataset for this specific GO term. The total # genes shows the total number of genes associated with that specific GO term. Nominal *p*-values and false discovery rate (FDR) *p*-values for the GO terms are also presented.

Supplementary Table 7 Significant biological processes (upregulated genes) for the M3 vs. M0 group comparison adjusted for sex, study, and differences in cell type composition (model 2). The column significant genes shows the number of significant genes identified from our dataset for this specific GO term. The total # genes shows the total number of genes associated with that specific GO term. Nominal *p*-values and false discovery rate (FDR) *p*-values for the GO terms are also presented.

Supplementary Table 8 Significant biological processes (downregulated genes) for the M3 vs. M0 group comparison adjusted for sex, study, and differences in cell type composition (model 2). The column significant genes shows the number of significant genes identified from our dataset for this specific GO term. The total # genes shows the total number of genes associated with that specific GO term. Nominal *p*-values and false discovery rate (FDR) *p*-values for the GO terms are also presented.

Supplementary Table 9 Significant pathways (upregulated genes) from the WikiPathways collection for the M3 vs. M0 group comparison adjusted for sex and study (model 1). The column significant genes shows the number of significant genes identified from our dataset for this pathway. The total # genes shows the total number of genes associated with that pathway. Nominal *p*-values and false discovery rate (FDR) *p*-values for the GO terms are also presented.

Supplementary Table 10 Significant pathways (downregulated genes) from the WikiPathways collection for the M3 vs. M0 group comparison adjusted for sex and study (model 1). The column significant genes shows the number of significant genes identified from our dataset for this pathway. The total # genes shows the total number of genes associated with that pathway. Nominal *p*-values and false discovery rate (FDR) *p*-values for the GO terms are also presented.

Supplementary Table 11 Significantly upregulated genes sensitivity analysis that excludes study GSE27916 for the M3 vs. M0 group comparison adjusted for sex and study (model 1). The log_2_ fold changes, nominal, and FDR *p*-values are shown.

Supplementary Table 12 Significantly downregulated genes sensitivity analysis that excludes study GSE27916 for the M3 vs. M0 group comparison adjusted for sex and study (model 1). The log_2_ fold changes, nominal, and FDR *p*-values are shown.

Supplementary Table 13 Significantly upregulated genes sensitivity analysis that excludes study E-MTAB-1985 for the M3 vs. M0 group comparison adjusted for sex and study (model 1). The log_2_ fold changes, nominal, and FDR *p*-values are shown.

Supplementary Table 14 Significantly downregulated genes sensitivity analysis that excludes study E-MTAB-1985 for the M3 vs. M0 group comparison adjusted for sex and study (model 1). The log_2_ fold changes, nominal, and FDR *p*-values are shown.

Supplementary Table 15 Significant biological processes (upregulated genes) for the M3 vs. M0 group comparison adjusted for sex and study (model 1), for the sensitivity analysis that excludes study GSE27916. The column significant genes shows the number of significant genes identified from our dataset for this specific GO term. The total # genes shows the total number of genes associated with that specific GO term. Nominal *p*-values and false discovery rate (FDR) *p*-values for the GO terms are also presented.

Supplementary Table 16 Significant biological processes (downpregulated genes) for the M3 vs. M30 group comparison adjusted for sex and study (model 1), for the sensitivity analysis that excludes study GSE27916. The column significant genes shows the number of significant genes identified from our dataset for this specific GO term. The total # genes shows the total number of genes associated with that specific GO term. Nominal *p*-values and false discovery rate (FDR) *p*-values for the GO terms are also presented.

Supplementary Table 17 Significant biological processes (upregulated genes) for the M3 vs. M0 group comparison adjusted for sex and study (model 1), for the sensitivity analysis that excludes study E-MTAB-1895. The column significant genes shows the number of significant genes identified from our dataset for this specific GO term. The total # genes shows the total number of genes associated with that specific GO term. Nominal *p*-values and false discovery rate (FDR) *p*-values for the GO terms are also presented.

Supplementary Table 18 Significant biological processes (downregulated genes) for the M3 vs. M0 group comparison adjusted for sex and study (model 1), for the sensitivity analysis that excludes study E-MTAB-1895. The column significant genes shows the number of significant genes identified from our dataset for this specific GO term. The total # genes shows the total number of genes associated with that specific GO term. Nominal *p*-values and false discovery rate (FDR) *p*-values for the GO terms are also presented.
