# Supplementary figures and images for: Assessing the Contribution of Relative Macrophage Frequencies to Subcutaneous Adipose Tissue
Source: Front Nutr. 2021 May 31;8:675935. doi: 10.3389/fnut.2021.675935 (PMC8200404; doi:10.3389/fnut.2021.675935)

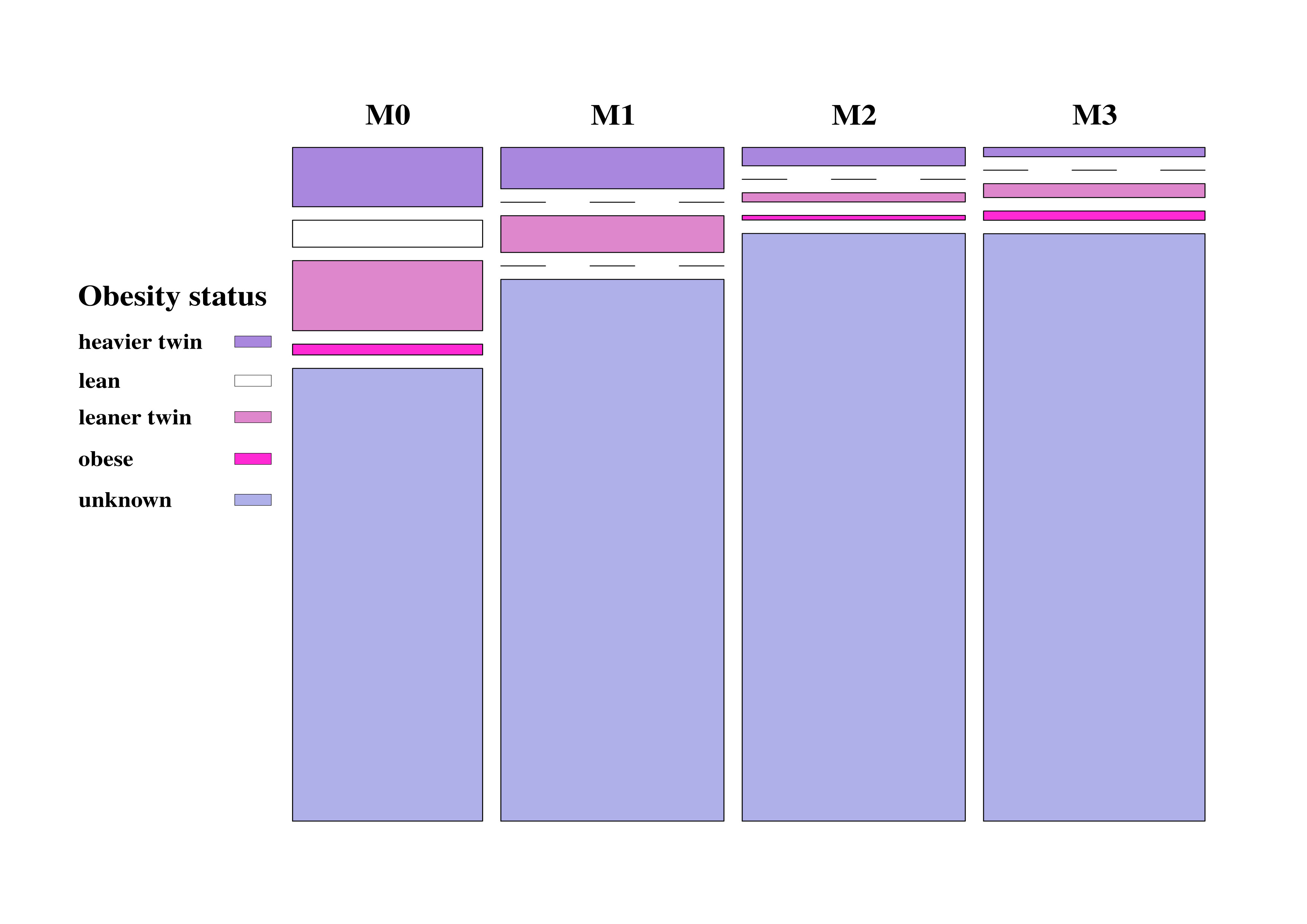

Supplement: Supplementary file 1 [file Data_Sheet_1.ZIP › Tables/Figure S1.jpg]

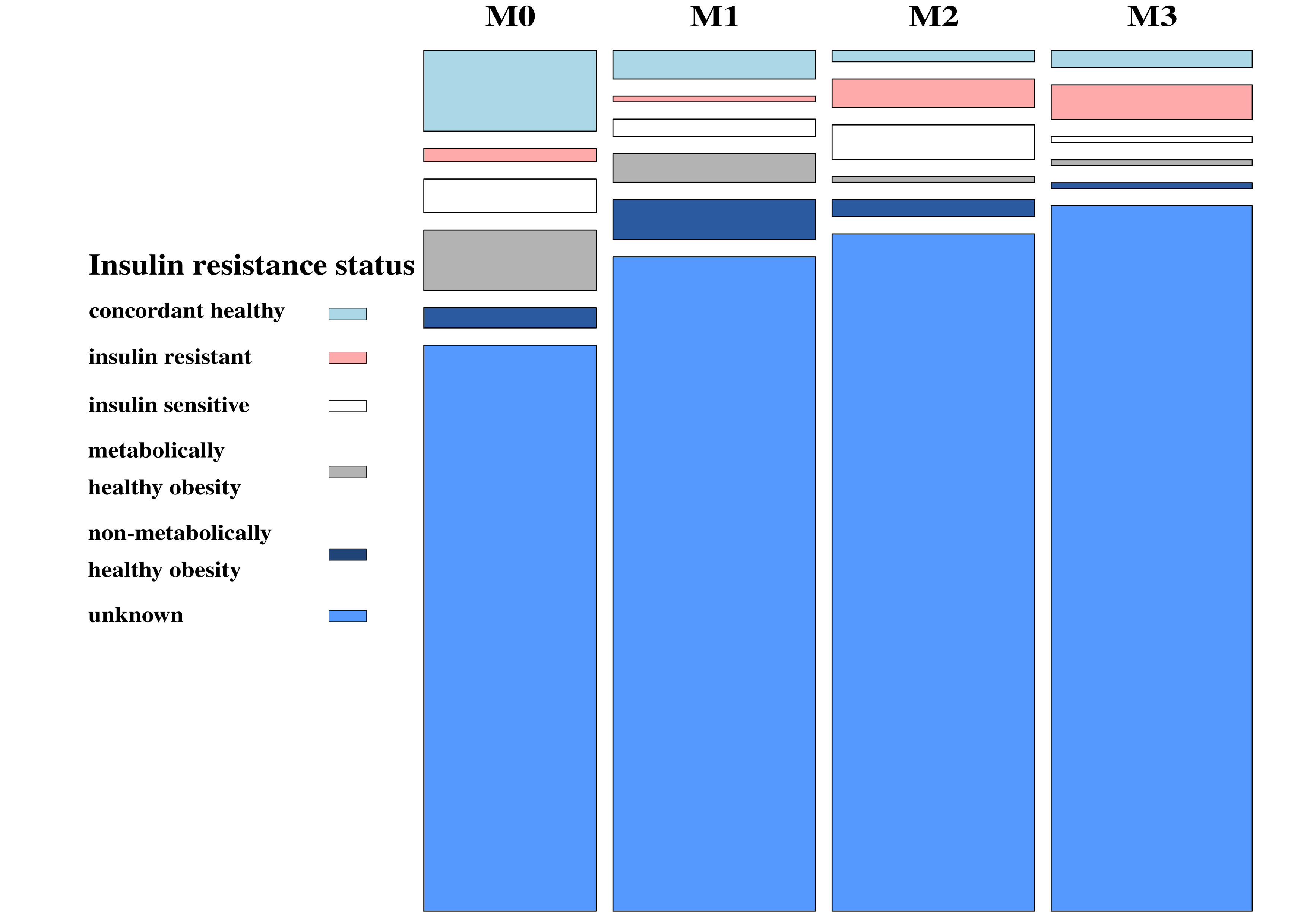

Supplement: Supplementary file 1 [file Data_Sheet_1.ZIP › Tables/Figure S2.jpg]
